# Supplementary material for: Online-Assisted Survey on Antibiotic Use by Pet Owners in Dogs and Cats
Source: Antibiotics (Basel). 2024 Apr 24;13(5):382. doi: 10.3390/antibiotics13050382 (PMC11117295; doi:10.3390/antibiotics13050382)
Supplement: Supplementary file 1 [file antibiotics-13-00382-s001.zip › Document S1-Pet owner questionnaire.pdf]

Mark as shown: ☐ ☒ ☐ ☐ ☐ Please use a ball-point pen or a thin felt tip. This form will be processed automatically.

Correction: ☐ ■ ☐ ☒ ☐ Please follow the examples shown on the left hand side to help optimize the reading results.

1. Information sheet

Dear dog/-cat owners,

you have a dog and/or a cat and have given an antibiotic to your pet within the last year? If that applies to you, we kindly ask you to share your experiences and opinions regarding the administration of antibiotics within the survey on "Antibiotic use by pet owners in dogs and cats", which I am conducting as part of my doctoral thesis at the Small Animal Medical Clinic of the Ludwig Maximilian University of Munich.

Antibiotics are important drugs for the treatment of infections in humans and animals; it is impossible to imagine veterinary and human medicine without them. Nevertheless, you have probably heard about the problem of antibiotic resistance, which leads to a loss of efficacy of these important drugs.

With this study we would like to gain new insights into the use of antibiotics by pet owners in dogs and cats. Therefore, if you have given your dog/cat an antibiotic within the last year, we would greatly appreciate it if you could take the time to participate in the survey. The completion of the anonymous questionnaire will take approximately 15 minutes.

This participation is voluntary and your information will be analysed statistically and anonymously. You can participate in the survey from both a computer and a mobile device.

People under the age of 18 and veterinarians are excluded from the survey. If you encounter any problems completing the questionnaire, please contact me via E-Mail (Clara Rocholl: clara.rocholl@campus.lmu.de).

Thank you very much for your support!

Clara Rocholl, doctoral student in veterinary medicine at the Medical Small Animal Clinic of the LMU Munich Email: clara.rocholl@campus.lmu.de

Bianka Schulz, private docent, Dr. med. vet, Dr. habil., Faculty at the Medical Small Animal Clinic of the LMU Munich Email: Bianka.Schulz@LMU.de

DRAFT

2. Declaration of consent for participation in the survey on the topic "Antibiotic use by pet owners in dogs and cats"

On the following pages we would like to ask you questions about the use of antibiotics. The survey is for dog and cat owners in Germany.
Within the scope of the survey, we also want to ask you for information about yourself in order to include social factors (age, gender, federal state, education) in the results and thus improve the evaluation.

We assure you, that all your answers will be treated absolutely anonymously and confidentially. Your answers will only be used for evaluation in the form of general statistical data.

Before you finally submit your answers, you have the option of viewing your answers again via the page navigation and changing them if necessary.

To enable easier readability of the survey, no gender-specific differentiation (e.g. veterinarian) was made. Where the masculine form is used in the following, all genders are equally addressed.

2.1

- ☐ Yes, I agree and would like to participate in the survey
- ☐ No I do not agree and do not wish to participate in the survey

DRAFT

3. Information about your pet

In the following we would like to ask you some questions about your pet and about the last antibiotic administration in this particular animal.

If you have more than one dog and/or cat that has received an antibiotic in the past, please answer the following questions for the animal that last received an antibiotic.

3.1 **Which animal is it?**

☐ Dog ☐ Cat

3.2 **Is it an outdoor cat?**

☐ Yes ☐ No

3.3 **How old is your dog/cat currently?**

☐ 0 - 3 months
☐ 4 - 6 months
☐ 7 - 9 months
☐ 10 - 11 months
☐ 1 year
☐ 2 years
☐ 3 years
☐ 4 years
☐ 5 years
☐ 6 years
☐ 7 years
☐ 8 years
☐ 9 years
☐ 10 years
☐ 11 years
☐ 12 years
☐ 13 years
☐ 14 years
☐ 15 years
☐ 16 years
☐ 17 years
☐ 18 years
☐ 19 years
☐ 20 years and older
☐ Fully grown but age unknown
☐ Unfortunately, my animal passed away in the meantime

3.4 **What gender is your animal?**

☐ Female ☐ Male ☐ Female neutered
☐ Male neutered

3.5 **What breed is your dog?**

☐ Airedale Terrier
☐ Akita Inu
☐ Australian Shepherd
☐ Basset
☐ Beagle
☐ Saint Bernard
☐ Bernese Mountain Dog
☐ Border Collie
☐ Boston Terrier
☐ Briard
☐ Bull Terrier
☐ Cavalier King Charles Spaniel
☐ Chihuahua
☐ Collie
☐ Dachshund
☐ Dalmatian
☐ Boxer
☐ Great Dane
☐ German Wirehaired Pointer
☐ Jagdterrier
☐ German Shorthaired Pointer
☐ German Shepherd
☐ Doberman
☐ Old English Bulldog
☐ English Cocker Spaniel
☐ French Bulldog
☐ Flat Coated Retriever
☐ Golden Retriever
☐ Havanese dog
☐ Hovawart
☐ Husky
☐ Irish Wolfhound
☐ Jack Russel Terrier
☐ Small Muensterlaender
☐ Labrador Retriever
☐ Leonberger
☐ Malinois
☐ Maltese dog
☐ Miniature Bull Terrier
☐ Standard Schnauzer
☐ Pug
☐ Newfoundland dog
☐ Parson Russel Terrier
☐ Poodle
☐ Rhodesian Ridgeback
☐ Giant Schnauzer
☐ Rottweiler
☐ Shar Pei
☐ Pomeranian
☐ Tibetan Terrier
☐ Weimeraner
☐ West Highland Terrier
☐ Yorkshire Terrier
☐ Miniature Schnauzer
☐ Other breed
☐ Mixed breed
☐ I do not know

3.6 **What other breed is it (if none of the above)?**

____

3. Information about your pet [Continue]

3.7 **What breed is your cat?**

- ☐ Abyssinian cat
- ☐ Bengal cat
- ☐ British Shorthair
- ☐ British Longhair
- ☐ Burmese cat
- ☐ Cornish Rex
- ☐ Domestic Longhair
- ☐ Devon Rex
- ☐ European Shorthair
- ☐ Exotic Shorthair
- ☐ German Rex
- ☐ Birman
- ☐ Chartreux
- ☐ Maine Coon
- ☐ Norwegian forest cat
- ☐ Persian
- ☐ Ragdoll
- ☐ Russian Blue
- ☐ Savannah cat
- ☐ Siamese cat
- ☐ Sphynx cat
- ☐ Turkish Angora
- ☐ Other breed
- ☐ Mixed breed
- ☐ I do not know

3.8 **What other breed is your cat (if none of the above)?**

3.9 **Does your pet have pet health insurance?** ☐ Yes ☐ No

4. Antibiotic administration in your pet

4.1 **What problem was the reason for the last antibiotic administration?**
(Multiple answers possible)

If you know the underlying disease of your animal for which the antibiotic was given, you are welcome to additionally mark "Diagnosis" and then write it in the field below this question.

- ☐ Diarrhea
- ☐ Vomiting
- ☐ Nasal discharge
- ☐ Infection of the upper respiratory tract (nose, paranasal sinuses, throat)
- ☐ Skin problems
- ☐ Ear infection
- ☐ Cough
- ☐ Infection of the lower respiratory tract (larynx, trachea, bronchi, lungs)
- ☐ Fever
- ☐ Wound infection/abscess/bite injury
- ☐ Castration/neutering
- ☐ Surgery of the bones
- ☐ Surgery on soft tissues (e.g. in the stomach or on the skin)
- ☐ Dental treatment
- ☐ Other surgery
- ☐ Urinary tract infection (kidney, bladder, prostate)
- ☐ Eye problem
- ☐ Other problem
- ☐ Diagnosis

4.2 **What other problem (besides the ones listed above) was the reason for the last antibiotic administration?**

4.3 **Which diagnosis was the reason for the last antibiotic administration?**

4.4 **When your dog/cat was last given an antibiotic, in what form did you give it?** (Multiple answers possible)

- ☐ Tablets
- ☐ Capsules
- ☐ Oral solution/suspension/paste
- ☐ Injections/Syringes
- ☐ Topical (ointment, eye/ear drops/ointment)

4.5 **Over what time period was the antibiotic administered**? (Please indicate the number of days)

- ☐ 1 day
- ☐ 2 days
- ☐ 3 days
- ☐ 4 days
- ☐ 5 days
- ☐ 6 days
- ☐ 7 days
- ☐ 8 days
- ☐ 9 days
- ☐ 10 days
- ☐ 11 days
- ☐ 12 days
- ☐ 13 days
- ☐ 14 days
- ☐ 15 days or longer
- ☐ I do not know anymore

4.6 **How often were you supposed to administer the antibiotic per day?**

- ☐ Once
- ☐ 2 times
- ☐ 3 times
- ☐ 4 times
- ☐ 5 times
- ☐ 6 times
- ☐ More than 6 times
- ☐ I do not know anymore

4.7 **What kind of injection was given?**

- ☐ Long-acting injection (effective up to 14 days)
- ☐ Short-acting injection (effective 1-2 days)
- ☐ I do not know anymore

4. Antibiotic administration in your pet [Continue]

4.8 **How often did your animal receive an injection with the antibiotic?** (during the last antibiotic administration)

- ☐ Once
- ☐ 2 times
- ☐ 3 times
- ☐ 4 times
- ☐ 5 times
- ☐ 6 times
- ☐ 7 times
- ☐ 8 times
- ☐ 9 times
- ☐ 10 times
- ☐ More than 10 times
- ☐ I do not know anymore

4.9 **Which antibiotic did your animal receive for the last administration?** (Multiple answers possible)

Note: The active ingredient or trade name can be found on the package of the antibiotic or on the invoice from your veterinarian. The antibiotic might also have been given to you with a bag labelled with the name of the antibiotic.

black = tablets/capsules
blue = injection/syringe
orange = oral solution/suspension/paste

- ☐ Ampicillin (e.g. Ampitab, Ampicillin-Dosierer, Albipen LA)
- ☐ Amoxicillin (e.g. Amox, Amoxicillin, Amoxitab, Amoxival Vet, Wedemox, Duphamox, Vetrimoxin-Paste)
- ☐ Amoxicillin/clavulanic acid (e. g. Synulox (injection/tablets), Clavaseptin, AmoxiClav, Clavudale, Clavucill, Kesium, Nicilan)
- ☐ Cefalexin (e.g. Cephacare, Rilexine, Therios, Cefaseptin, Cefatab, Tsefalen)
- ☐ Cefazolin (e.g. Cefazolin)
- ☐ Cefovecin (e.g. Convenia (effective up to 14 days))
- ☐ Clindamycin (e.g. Clindamycin, Aniclindan, Cleorobe, Zodon, Clindaseptin (capsule/ solution), Clindabactin)
- ☐ Doxycycline (e.g. Ronaxan, Doxybactin, Doxytab)
- ☐ Enrofloxacin (e.g. Baytril (injection/tablets), Enrotab, Xeden, Enrofloxacin (injection/ tablets), Enrox Flavour, Zobuxa)
- ☐ Gentamicin (e.g. Genta 100, Genta 5%)
- ☐ Marbofloxacin (e.g. Marbocyl (injection/tablets), Marbotab, Quiflor (injection/tablets), Efex)
- ☐ Metronidazole (e.g. Metronidazol, Metrobactin, Metrivis, Suanatem, Spizobactin, Eradia)
- ☐ Penicillin (e.g. Belamycin, Procillin)
- ☐ Pradofloxacin (e.g. Veraflox (tablets/suspension)
- ☐ TSO (e.g. Sulfadimidin, TSO-Tabletten, Forthoprim)
- ☐ Other antibiotic
- ☐ I do not know anymore

4. Antibiotic administration in your pet [Continue]

4.10 **Which antibiotic did you use topically in your animal at the last administration?**
(Multiple answers possible)

Note: The active ingredient or trade name can be found on the package of the antibiotic or on the invoice from your veterinarian. The antibiotic might also have been given to you with a bag labelled with the name of the antibiotic.

red = topical skin/ear (OT = eardrops)
green = topical eye (AS = eye ointment, AT = eye drops)

- ☐ Chloramphenicol (e.g. Cefenicol CA AT, Posifenicol AS, Cefenidex AT, Chloramphenicol-Pumpspray, Prurivet)
- ☐ Chlortetracycline (e.g. Cepemycin AS, Ophtocycline AS)
- ☐ Florfenicol (e.g. Osurnia Ohrengel)
- ☐ Fusidic acid (e.g. Isaderm, Isathal AT)
- ☐ Gentamicin (e.g. Otomax OT, Easotic OT, Ophtogent Augengel, Soligental AT)
- ☐ Moxifloxacin (e.g. Vigamox AT)
- ☐ Neomycin (e.g. Dermamycin (AS/skin creme)
- ☐ Marbofloxacin (e.g. Aurizon OT)
- ☐ Oxytetracycline (e.g. Oxytetracyclin AS)
- ☐ Polymyxin, neomycin sulfate, gramicidin (e.g. Polyspectran AT)
- ☐ Polymyxin (e.g. Surolan OT, Ototob OT, Mitex OT)
- ☐ Ofloxacin (e.g. Floxal AT/AS)
- ☐ Other antibiotic
- ☐ I do not know anymore

4.11 **Which other antibiotic has your animal received?** (Please indicate the name of the preparation or the active ingredient)

[ ]

4.12 **Did you expect the prescription of an antibiotic for this problem of your animal?**

- ☐ Yes
- ☐ No

4. Antibiotic administration in your pet [Continue]

Please rate the following statements in relation to the last antibiotic administration in your animal.

	Strongly disagree	Disagree	Neutral	Agree	Strongly agree	I do not know
4.13 My veterinarian involved and advised me in the decision of the choice of the form of administration (tablets, liquid, injections, etc.)	☐	☐	☐	☐	☐	☐
4.14 I would have liked more education from my vet on how to best insert/use the antibiotic (for example, tablet insertion with food/treat/tablet dispenser)	☐	☐	☐	☐	☐	☐
4.15 My veterinarian has informed me about possible side effects	☐	☐	☐	☐	☐	☐
4.16 My vet told me exactly how to give the antibiotic (for example, how often, how many tablets/injections, how long)	☐	☐	☐	☐	☐	☐
4.17 I followed exactly the recommendations of my veterinarian for the antibiotic administration	☐	☐	☐	☐	☐	☐
4.18 I stuck to the exact number of tablets/ capsules per antibiotic administration	☐	☐	☐	☐	☐	☐
4.19 I stuck to the exact amount of solution/suspension/paste to be given per antibiotic administration	☐	☐	☐	☐	☐	☐
4.20 I followed the exact number of antibiotic doses per day	☐	☐	☐	☐	☐	☐
4.21 I strictly followed the time intervals between antibiotic administrations	☐	☐	☐	☐	☐	☐
4.22 I have suspended the administration of the antibiotic at least once because I was not able to administer it	☐	☐	☐	☐	☐	☐
4.23 I always went to the vet for the injections on the scheduled appointment	☐	☐	☐	☐	☐	☐
4.24 I skipped an injection at least once and did not go to the vet	☐	☐	☐	☐	☐	☐

DRAFT

4. Antibiotic administration in your pet [Continue]

	Not at all	Rather less good	Neutral	Rather good	Very good (without problems)
4.25 **How well were you able to give your animal the antibiotic?**	☐	☐	☐	☐	☐
4.26 **How well were you able to apply the antibiotic topically to your animal?**	☐	☐	☐	☐	☐

4.27 **How did you give the tablets/capsules to your animal?** (Multiple answers possible)
- ☐ With food
- ☐ Hidden in food/treats
- ☐ With tablet dispenser
- ☐ With special treat, trojaner (e. g. EasyPill®, Pill Assist™ by Royal Canin)
- ☐ Crushed tablets to powder and mixed with food/treats
- ☐ Crushed tablets to powder and injected into mouth with liquid via syringe
- ☐ Capsule content given without capsule
- ☐ Given directly into the mouth
- ☐ My animal ate the tablets/ capsules out of hand without any additional aids
- ☐ Other

4.28 **Did you wash your hands immediately after administering the antibiotic?**
- ☐ Yes
- ☐ No
- ☐ No, but I wore gloves
- ☐ Yes and I wore gloves
- ☐ I do not know anymore

4.29 **Did you stop antibiotic administration before the duration of use indicated by your veterinarian was completed?**
- ☐ Yes
- ☐ No
- ☐ I do not know anymore

4.30 **What were the reasons for stopping the administration early?** (Multiple answers possible)
- ☐ Difficulties with ingestion/ application
- ☐ Lack of time
- ☐ Side effects have occurred
- ☐ Fear of potential side effects
- ☐ My dog/cat had already recovered
- ☐ Consultation with my veterinarian
- ☐ Other reason

4.31 **Did your animal experience any side effects that may have occurred as a result of the administration of the antibiotic?**
- ☐ Yes
- ☐ No
- ☐ I do not know

DRAFT

4. Antibiotic administration in your pet [Continue]

4.32 **What side effects have occurred?** (Multiple answers possible)

- ☐ Diarrhea
- ☐ Vomiting
- ☐ Allergic reaction
- ☐ Other side effect

5. Test before the use of an antibiotic

Please also refer to your pet's <u>last</u> antibiotic administration when answering the following questions.

5.1 **Did your veterinarian perform any tests prior to administering the antibiotic, based on the results of which the antibiotic was prescribed/administered?** (Multiple answers possible)

- ☐ No
- ☐ Yes, there were changes in the blood count
- ☐ Yes, there were changes in the X-ray images
- ☐ Yes, changes were present in the urine analysis
- ☐ Yes, changes were present in the ultrasound examination
- ☐ Yes, changes were present in a microscopic examination
- ☐ Yes, changes were present in other examinations
- ☐ Yes, but I do not know what test or changes exactly
- ☐ I do not know

5.2 **Was a culture with resistance test or other tests for pathogen detection performed prior to antibiotic administration/prescription?** These tests can be used to investigate whether pathogens are present for which an antibiotic is necessary, and which antibiotic exactly is effective.

- ☐ Yes
- ☐ No
- ☐ I do not know

6. Preferred route of administration

What is your preferred route of administration for systemic antibiotic administration (not applied locally) in your animal?

Please tick how much you prefer each possible form of administration.

		Not at all	Little	Neutral	Strong	Very strong
6.1	Tablets	☐	☐	☐	☐	☐
6.2	Capsules	☐	☐	☐	☐	☐
6.3	Oral solution/suspension/paste	☐	☐	☐	☐	☐
6.4	Short-acting injection (effective 1-2 days) - multiple injections often necessary	☐	☐	☐	☐	☐
6.5	Long-acting injection (one injection effective up to 14 days)	☐	☐	☐	☐	☐

		Not at all	Rather less good	Neutral	Rather good	Very good	I have not had to give any so far
6.6	**How well does your dog/cat take tablets?**	☐	☐	☐	☐	☐	☐

6.7 **Is your pet's ability to take tablets related to the taste of the medication?**

☐ Yes ☐ No ☐ I do not know

6.8 **Does your dog/cat tolerate liquid/paste medication (solution/suspension/paste) better than tablets/capsules?**

☐ Yes ☐ No ☐ I do not know

7. Choosing an antibiotic

Please rate how important the following are to you when choosing an antibiotic.

		Not at all important	Not very important	Somewhat important	Very important	Extremely important
7.1	Easy form of administration	☐	☐	☐	☐	☐
7.2	Costs	☐	☐	☐	☐	☐
7.3	Tolerability (potential side effects)	☐	☐	☐	☐	☐
7.4	Avoiding the development of antibiotic resistance	☐	☐	☐	☐	☐
7.5	Efficacy	☐	☐	☐	☐	☐
7.6	Recommendation of my veterinarian	☐	☐	☐	☐	☐
7.7	Tastefulness of the antibiotic	☐	☐	☐	☐	☐
7.8	Avoidance of "reserve antibiotics" (antibiotics used to treat resistant pathogens. Use as "last resort" for people with infections with antibiotic-resistant pathogens)	☐	☐	☐	☐	☐

8. Demographics

This last section contains questions about you as a pet owner. This information helps us to compare your experiences and opinions regarding the administration of antibiotics to your animal within the different groups of people and regions in Germany. Providing your data is voluntary and we will not use your data for any other purpose. If you do not wish to answer a question, you can simply skip it and carry on to the next question.

8.1 **What is your gender?** ☐ Female ☐ Male ☐ Diverse

8.2 **How old are you?** (Age in years)

☐ 18	☐ 19	☐ 20
☐ 21	☐ 22	☐ 23
☐ 24	☐ 25	☐ 26
☐ 27	☐ 28	☐ 29
☐ 30	☐ 31	☐ 32
☐ 33	☐ 34	☐ 35
☐ 36	☐ 37	☐ 38
☐ 39	☐ 40	☐ 41
☐ 42	☐ 43	☐ 44
☐ 45	☐ 46	☐ 47
☐ 48	☐ 49	☐ 50
☐ 51	☐ 52	☐ 53
☐ 54	☐ 55	☐ 56
☐ 57	☐ 58	☐ 59
☐ 60	☐ 61	☐ 62
☐ 63	☐ 64	☐ 65
☐ 66	☐ 67	☐ 68
☐ 69	☐ 70	☐ 71
☐ 72	☐ 73	☐ 74
☐ 75	☐ 76	☐ 77
☐ 78	☐ 79	☐ 80
☐ 81	☐ 82	☐ 83
☐ 84	☐ 85	☐ 86
☐ 87	☐ 88	☐ 89
☐ 90	☐ Older than 90	

8.3 **In which federal state do you live in?**

☐ Baden-Wuerttemberg	☐ Bavaria	☐ Berlin
☐ Brandenburg	☐ Bremen	☐ Hamburg
☐ Hesse	☐ Mecklenburg-Western Pomerania	☐ Lower Saxony
☐ North-Rhine Westphalia	☐ Rhineland-Palatine	☐ Saarland
☐ Saxon	☐ Saxony-Anhalt	☐ Schleswig-Holstein
☐ Thuringia		

8.4 **Do you have any medical training?** ☐ Yes ☐ No

8.5 **In what field do you have the medical training?** ☐ Veterinary Medicine ☐ Human Medicine

8. Demographics [Continue]

8.6 **What is your highest level of education?**

- ☐ No degree (yet)
- ☐ Lower Certificate of Secondary Education (Hauptschulabschluss, qualifizierender Hauptschulabschluss)
- ☐ Intermediate Certificate of Secondary Education (Realschulabschluss, Mittlerer Bildungsabschluss)
- ☐ Higher Education Entrance Qualification (Fachhochschulreife (Allgemeine oder fachgebundene Fachhochschulreife))
- ☐ High School Diploma (Abitur (Allgemeine oder fachgebundene Hochschulreife))
- ☐ Vocational Training (Berufsausbildung)
- ☐ University of Applied Sciences degree (Fachhochschulabschluss (Bachelor, Master, Diplom))
- ☐ University degree (Hochschulabschluss (Bachelor, Master, Staatsexamen, Diplom, Magister, Promotion))
- ☐ Other

DRAFT

9. Thank you very much!

Thank you for your support! And please do not forget to click "Submit" :)

9.1 **Do you have any suggestions or comments about this survey?**

DRAFT
