# Supplementary material for: Online-Assisted Survey on Antibiotic Use by Pet Owners in Dogs and Cats
Source: Antibiotics (Basel). 2024 Apr 24;13(5):382. doi: 10.3390/antibiotics13050382 (PMC11117295; doi:10.3390/antibiotics13050382)
Supplement: Supplementary file 1 [file antibiotics-13-00382-s001.zip › Document S2-Supplementary Data .pdf]

Supplementary Data

Table S1. Pet owner demographics.

**Gender**	Female	676/705 (95.9%)
	Male	25/705 (3.5%)
	Diverse	4/705 (0.6%)
**Age Median (IQR)**		39 (30, 50)
**Federal state**	Baden-Wuerttemberg	71/693 (10.2%)
	Bavaria	213/693 (30.7%)
	Berlin	11/693 (1.6%)
	Brandenburg	15/693 (2.2%)
	Bremen	9/693 (1.3%)
	Hamburg	9/693 (1.3%)
	Hesse	44/693 (6.3%)
	Mecklenburg-Western Pomerania	11/693 (1.6%)
	Lower Saxony	59/693 (8.5%)
	North-Rhine Westphalia	150/693 (21.6%)
	Rhineland-Palatine	29/693 (4.2%)
	Saarland	7/693 (1.0%)
	Saxon	13/693 (1.9%)
	Saxony-Anhalt	11/693 (1.6%)
	Schleswig-Holstein	35/693 (5.1%)
	Thuringia	6/693 (0.9%)
**Medical Training**	Yes	206/704 (29.3%)
	No	498/704 (70.7%)
**Field of medical education**	Veterinary Medicine	93/205 (45.4%)
	Human Medicine	112/205 (54.6%)

**Comparison of educational level with medical education**	Overall n = 706	Medical Training n = 206	No Medical Training n = 498	Veterinary Medicin n = 93	Human Medicin n = 112
No degree (yet)	2 (0.3%)	1 (0.5%)	1 (0.2%)	1 (1.1%)	0 (0.0%)
Lower Certificate of Secondary Education (Hauptschulabschluss)	9 (1.3%)	0 (0.0%)	9 (1.8%)	0 (0.0%)	0 (0.0%)
Intermediate Certificate of Secundary Education (Realschulabschluss)	71 (10.1%)	19 (9.3%)	52 (10.5%)	6 (6.5%)	13 (11.6%)
Higher Education Entrance Qualification (Fachhochschulreife)	48 (6.8%)	13 (6.3%)	34 (6.8%)	5 (5.4%)	7 (6.2%)
High School Diploma (Abitur)	102 (14.4%)	37 (18.0%)	65 (13.1%)	23 (25.0%)	14 (12.5%)
Vocational Training (Berufsausbildung)	194 (27.5%)	76 (37.1%)	118 (23.7%)	29 (31.5%)	47 (42.0%)
University of Applied Sciences degree (Fachhochschulabschluss)	72 (10.2%)	9 (4.4%)	63 (12.7%)	2 (2.2%)	7 (6.2%)
University degree (Hochschulabschluss)	201 (28.5%)	47 (22.9%)	151 (30.4%)	23 (25.0%)	24 (21.4%)
Other	7 (1.0%)	3 (1.5%)	4 (0.8%)	3 (3.3%)	(0.0%)

Table S2. Demographic data of dogs and cats.

**Animal species**	Dog	463/708 (65.4%)	
	Cat	245/708 (34.6%)	
**Outdoor cat**	Yes	105/245 (42.9%)	
	No	140/245 (57,1%)	
**Breed**	Purebred cat	210/239 (87.9%)	
	Mixed breed cat	29/239 (12.1%)	
	Purebred dog	333/462 (72.1%)	
	Mixed breed dog	129/462 (27.9%)	
**Gender**	**Overall, n = 708**	**Dog, n = 463**	**Cat, n = 245**
Female	144 (20.3%)	115 (24.8%)	29 (11.8%)
Male	129 (18.2%)	111 (24.0%)	18 (7.3%)
Female neutered	205 (29.0%)	120 (25.9%)	85 (34.7%)
Male neutered	230 (32.5%)	117 (25.3%)	113 (46.1%)
**Age**	**Overall, n = 708**	**Dog, n = 463**	**Cat, n = 245**
0-3 months	1 (0.1%)	1 (0.2%)	0 (0.0%)
4-6 months	11 (1.6%)	7 (1.5%)	4 (1.6%)
7-9 months	12 (1.7%)	8 (1.7%)	4 (1.6%)
10-11 months	12 (1.7%)	8 (1.7%)	4 (1.6%)
1 year	63 (8.9%)	42 (9.1%)	21 (8.6%)
2 years	84 (11.9%)	57 (12.3%)	27 (11.0%)
3 years	84 (11.9%)	49 (10.6%)	35 (14.3%)
4 years	59 (8.3%)	38 (8.2%)	21 (8.6%)
5 years	51 (7.2%)	36 (7.8%)	15 (6.1%)
6 years	53 (7.5%)	44 (9.5%)	9 (3.7%)
7 years	29 (4.1%)	24 (5.2%)	5 (2.0%)
8 years	37 (5.2%)	22 (4.8%)	15 (6.1%)
9 years	38 (5.4%)	26 (5.6%)	12 (4.9%)
10 years	40 (5.6%)	26 (5.6%)	14 (5.7%)
11 years	27 (3.8%)	19 (4.1%)	8 (3.3%)
12 years	31 (4.4%)	22 (4.8%)	9 (3.7%)
13 years	18 (2.5%)	12 (2.6%)	6 (2.4%)
14 years	15 (2.1%)	9 (1.9%)	6 (2.4%)
15 years	11 (1.6%)	6 (1.3%)	5 (2.0%)
16 years	9 (1.3%)	2 (0.4%)	7 (2.9%)
17 years	5 (0.7%)	1 (0.2%)	4 (1.6%)
18 years	4 (0.6%)	0 (0.0%)	4 (1.6%)
19 years	0	0	0
20 years and older	3 (0.4%)	0 (0.0%)	3 (1.2%)
Fully grown but age unknown	2 (0.3%)	1 (0.2%)	1 (0.4%)
Unfortunately, my animal passed away in the meantime	9 (1.3%)	3 (0.6%)	6 (2.4%)
**Age Median (IQR)**		5 (3, 9)	5 (3, 10)

Table S3. Comparison of pet health insurance between dogs and cats.

	Overall n = 708	Dog n = 463	Cat n = 245	p-value[1]	q-value[2]	Cramer´s V[3]	95% CI[4]
Pet health insurance				< 0.001	< 0.001	0.13	0.07-1.00
Yes	174 (24.6%)	134 (28.9%)	40 (16.3%)				
No	534 (75.4%)	329 (71.1%)	205 (83.7%)				

[1]Pearson's Chi-squared test
[2]False discovery rate correction for multiple testing
[3]Interpretation: > 0.25 very strong, > 0.15 strong, > 0.10 moderate, > 0.05 weak, > 0 no or very weak association [27]
[4] CI = confidence interval of effect size

Antibiotic administration

Table S4. Owner expectation of antibiotic administration for different indications in dogs and cats.

Indication	Administration was expected n = 580	Administration was not expected n = 128	p-value[1]	q-value[2]	Cramer´s V[3]	95% CI[4]
Diarrhea (n = 107)	78 (72.9%)	29 (27.1%)	0.008	0.111	0.09	0.00-1.00
Vomiting (n = 59)	43 (72.9%)	16 (27.1%)	0.060	0.190	0.06	0.00-1.00
Infection of the upper respiratory tract (n = 63)	52 (82.5%)	11 (17.5%)	0.894	0.894	0.00	0.00-1.00
Skin problems (n = 50)	39 (78.0%)	11 (22.0%)	0.455	0.662	0.00	0.00-1.00
Ear infection (n = 50)	36 (72.0%)	14 (28.0%)	0.059	0.190	0.06	0.00-1.00
Infection of the lower respiratory tract (n = 54)	45 (83.3%)	9 (16.7%)	0.779	0.890	0.00	0.00-1.00
Fever (n = 47)	39 (83.0%)	8 (17.0%)	0.845	0.894	0.00	0.00-1.00
Wound infection/abscess/bite injury (n = 131)	111 (84.7%)	20 (15.3%)	0.354	0.567	0.00	0.00-1.00
Castration/neutering (n = 42)	40 (95.2%)	2 (4.8%)	0.021	0.111	0.08	0.00-1.00
Surgery of the bones (n = 28)	25 (89.3%)	3 (10.7%)	0.301	0.536	0.01	0.00-1.00
Surgery on soft tissue (n = 69)	64 (92.8%)	5 (7.2%)	0.014	0.111	0.08	0.00-1.00
Dental treatment (n = 90)	76 (84.4%)	14 (15.6%)	0.506	0.674	0.00	0.00-1.00
Other surgery (n = 12)	11 (91.7%)	1 (8.3%)	0.704	0.866	0.00	0.00-1.00
Urinary tract infection (n = 74)	64 (86.5%)	10 (13.5%)	0.281	0.536	0.02	0.00-1.00
Eye problem (n = 34)	24 (70.6%)	10 (29.4%)	0.078	0.209	0.05	0.00-1.00
Other problem (n = 97)	74 (76.3%)	23 (23.7%)	0.121	0.276	0.04	0.00-1.00

[1]Pearson's Chi-squared test; Fisher's exact test
[2]False discovery rate correction for multiple testing
[3]Interpretation: > 0.25 very strong, > 0.15 strong, > 0.10 moderate, > 0.05 weak, > 0 no or very weak association [27]
[4] CI = confidence interval of effect size

Table S5. Duration and frequency of orally or locally administered antibiotics.

**Over what time period was the antibiotic administered?**	
1 day	3/633 (0.5%)
2 days	3/633 (0.5%)
3 days	20/633 (3.2%)

4 days	13/633 (2.1%)
5 days	119/633 (18.8%)
6 days	22/633 (3.5%)
7 days	148/633 (23.4%)
8 days	19/633 (3.0%)
9 days	3/633 (0.5%)
10 days	132/633 (20.9%)
11 days	3/633 (0.5%)
12 days	14/633 (2.2%)
13 days	0/633 (0.0%)
14 days	50/633 (7.9%)
15 days or longer	84/633 (13.3%)
**How often were you supposed to administer the antibiotic per day?**	
Once	203/625 (32.5%)
2 times	388/625 (62.1%)
3 times	29/625 (4.6%)
4 times	2/625 (0.3%)
5 times	1/625 (0.2%)
6 times	1/625 (0.2%)
More than 6 times	1/625 (0.2%)

Table S6. Comparison between the type of injection and the number of injections administered.

	**Overall n = 155**	**Long-acting injection n = 30**	**Short-acting injection n = 125**	**p-value[1]**	**q-value[2]**	**Cramer´s V[3]**	**95% CI[4]**
Number of injections				0.315	0.315	0.16	0.00-1.00
Once	77 (49.7%)	17 (56.7%)	60 (48.0%)				
2 times	36 (23.2%)	12 (40.0%)	24 (19.2%)				
3 times	17 (11.0%)	1 (3.3%)	16 (12.8%)				
4 times	4 (2.6%)	0 (0.0%)	4 (3.2%)				
5 times	6 (3.9%)	0 (0.0%)	6 (4.8%)				
6 times	2 (1.3%)	0 (0.0%)	2 (1.6%)				
7 times	5 (3.2%)	0 (0.0%)	5 (4.0%)				
8 times	1 (0.6%)	0 (0.0%)	1 (0.8%)				
9 times	0 (0.0%)	0 (0.0%)	0 (0.0%)				
10 times	4 (2.6%)	0 (0.0%)	4 (3.2%)				
More than 10 times	3 (1.9%)	0 (0.0%)	3 (2.4%)				

[1]Fisher's exact test
[2]False discovery rate correction for multiple testing
[3]Interpretation: > 0.25 very strong, > 0.15 strong, > 0.10 moderate, > 0.05 weak, > 0 no or very weak association [27]
[4] CI = confidence interval of effect size

Table S7. Systemic antibiotic administration for the different indications in dogs and cats.

Indication	Ampicillin n = 2	Amoxicillin n = 101	Amoxicillin/Clavulanic acid n = 221	Cefalexin n = 12	Cefazolin n = 1	Cefovecin n = 13	Clindamycin n = 18	Doxycycline n = 46	Enrofloxacin n = 18
Diarrhea	1 (50.0%)	19 (18.8%)	25 (11.3%)	2 (16.7%)	0 (0.0%)	0 (0.0%)	3 (16.7%)	3 (6.5%)	3 (16.7%)
Vomiting	1 (50.0%)	16 (15.8%)	15 (6.8%)	0 (0.0%)	0 (0.0%)	0 (0.0%)	1 (5.6%)	1 (2.2%)	2 (11.1%)
Infection of the upper respiratory tract	0 (0.0%)	7 (6.9%)	26 (11.8%)	0 (0.0%)	0 (0.0%)	1 (7.7%)	0 (0.0%)	10 (21.7%)	2 (11.1%)
Skin problems	0 (0.0%)	11 (10.9%)	15 (6.8%)	2 (16.7%)	0 (0.0%)	2 (15.4%)	4 (22.2%)	2 (4.3%)	1 (5.6%)
Ear infection	0 (0.0%)	6 (5.9%)	7 (3.2%)	0 (0.0%)	0 (0.0%)	1 (7.7%)	3 (16.7%)	2 (4.3%)	3 (16.7%)
Infection of the lower respiratory tract	0 (0.0%)	9 (8.9%)	18 (8.1%)	0 (0.0%)	0 (0.0%)	0 (0.0%)	0 (0.0%)	12 (26.1%)	2 (11.1%)
Fever	0 (0.0%)	8 (7.9%)	17 (7.7%)	0 (0.0%)	0 (0.0%)	1 (7.7%)	0 (0.0%)	6 (13.0%)	3 (16.7%)
Wound infection/abscess/bite injury	1 (50.0%)	20 (19.8%)	48 (21.7%)	3 (25.0%)	1 (100.0%)	1 (7.7%)	2 (11.1%)	5 (10.9%)	0 (0.0%)
Castration/neutering	0 (0.0%)	5 (5.0%)	16 (7.2%)	2 (16.7%)	0 (0.0%)	0 (0.0%)	0 (0.0%)	1 (2.2%)	0 (0.0%)
Surgery of the bones	0 (0.0%)	4 (4.0%)	11 (5.0%)	3 (25.0%)	0 (0.0%)	0 (0.0%)	1 (5.6%)	0 (0.0%)	0 (0.0%)
Surgery on soft tissue	0 (0.0%)	18 (17.8%)	25 (11.3%)	0 (0.0%)	0 (0.0%)	1 (7.7%)	1 (5.6%)	3 (6.5%)	2 (11.1%)
Dental treatment	0 (0.0%)	14 (13.9%)	33 (14.9%)	1 (8.3%)	0 (0.0%)	5 (38.5%)	6 (33.3%)	6 (13.0%)	3 (16.7%)
Other surgery	0 (0.0%)	2 (2.0%)	5 (2.3%)	1 (8.3%)	0 (0.0%)	0 (0.0%)	0 (0.0%)	0 (0.0%)	0 (0.0%)
Urinary tract infection	0 (0.0%)	11 (10.9%)	23 (10.4%)	0 (0.0%)	0 (0.0%)	4 (30.8%)	0 (0.0%)	1 (2.2%)	5 (27.8%)
Eye problem	0 (0.0%)	2 (2.0%)	4 (1.8%)	0 (0.0%)	0 (0.0%)	0 (0.0%)	0 (0.0%)	5 (10.9%)	0 (0.0%)
Other problem	0 (0.0%)	9 (8.9%)	30 (13.6%)	0 (0.0%)	0 (0.0%)	1 (7.7%)	5 (27.8%)	16 (34.8%)	2 (11.1%)

	Gentamicin n = 1	Marbofloxacin n = 15	Metronidazole n = 47	Penicillin n = 2	Pradofloxacin n = 2	Trimethoprim and Sulfonamides (TSO) n = 6	Other n = 7	Unknown n = 242
Diarrhea	1 (100.0%)	2 (13.3%)	23 (48.9%)	0 (0.0%)	0 (0.0%)	2 (33.3%)	1 (14.3%)	41 (16.9%)
Vomiting	0 (0.0%)	0 (0.0%)	9 (19.1%)	0 (0.0%)	0 (0.0%)	2 (33.3%)	1 (14.3%)	25 (10.3%)
Infection of the upper respiratory tract	0 (0.0%)	1 (6.7%)	2 (4.3%)	0 (0.0%)	0 (0.0%)	0 (0.0%)	1 (14.3%)	20 (8.3%)
Skin problems	0 (0.0%)	1 (6.7%)	2 (4.3%)	0 (0.0%)	0 (0.0%)	0 (0.0%)	0 (0.0%)	16 (6.6%)
Ear infection	0 (0.0%)	1 (6.7%)	1 (2.1%)	0 (0.0%)	0 (0.0%)	0 (0.0%)	1 (14.3%)	20 (8.3%)
Infection of the lower respiratory tract	0 (0.0%)	2 (13.3%)	3 (6.4%)	0 (0.0%)	0 (0.0%)	1 (16.7%)	2 (28.6%)	14 (5.8%)
Fever	0 (0.0%)	1 (6.7%)	4 (8.5%)	0 (0.0%)	0 (0.0%)	2 (33.3%)	0 (0.0%)	17 (7.0%)
Wound infection/abscess/bite injury	0 (0.0%)	3 (20.0%)	3 (6.4%)	1 (50.0%)	1 (50.0%)	0 (0.0%)	0 (0.0%)	53 (21.9%)

	Gentamicin n = 1	Marbofloxacin n = 15	Metronidazole n = 47	Penicillin n = 2	Pradofloxacin n = 2	Trimethoprim and Sulfonamides (TSO) n = 6	Other n = 7	Unknown n = 242
Castration/neutering	0 (0.0%)	1 (6.7%)	7 (14.9%)	0 (0.0%)	0 (0.0%)	1 (16.7%)	1 (14.3%)	17 (7.0%)
Surgery of the bones	0 (0.0%)	0 (0.0%)	0 (0.0%)	0 (0.0%)	0 (0.0%)	1 (16.7%)	0 (0.0%)	10 (4.1%)
Surgery on soft tissue	0 (0.0%)	3 (20.0%)	3 (6.4%)	1 (50.0%)	0 (0.0%)	1 (16.7%)	0 (0.0%)	22 (9.1%)
Dental treatment	0 (0.0%)	2 (13.3%)	3 (6.4%)	1 (50.0%)	0 (0.0%)	0 (0.0%)	0 (0.0%)	29 (12.0%)
Other surgery	0 (0.0%)	0 (0.0%)	0 (0.0%)	0 (0.0%)	0 (0.0%)	0 (0.0%)	1 (14.3%)	4 (1.7%)
Urinary tract infection	0 (0.0%)	4 (26.7%)	2 (4.3%)	0 (0.0%)	0 (0.0%)	2 (33.3%)	1 (14.3%)	30 (12.4%)
Eye problem	0 (0.0%)	0 (0.0%)	4 (8.5%)	1 (50.0%)	0 (0.0%)	0 (0.0%)	3 (42.9%)	5 (2.1%)
Other problem	0 (0.0%)	1 (6.7%)	13 (27.7%)	0 (0.0%)	1 (50.0%)	2 (33.3%)	1 (14.3%)	26 (10.7%)

Table S8. 5-point Likert scale displaying veterinary education of pet owners and owner compliance with antibiotic administration. Comparison of data with medical training or no medical training.

	Overall n = 708	Medical training n = 206	No medical training n = 498	p-value[1]	q-value[2]	Cramer´s V[3]	95% CI[4]
**My veterinarian involved and advised me in the decision of the choice of the form of administration (tablets, liquid, injections, etc.) (n = 701/708)**				0.001	0.007	0.14	0.02-1.00
Strongly disagree	219 (31.2%)	44 (21.5%)	175 (35.6%)				
Disagree	58 (8.3%)	14 (6.8%)	44 (8.9%)				
Neutral	73 (10.4%)	21 (10.2%)	52 (10.6%)				
Agree	92 (13.1%)	31 (15.1%)	58 (11.8%)				
Strongly agree	259 (36.9%)	95 (46.3%)	163 (33.1%)				
**I would have liked more education from my vet on how to best insert/use the antibiotic (for example, tablet insertion with food/treat/tablet dispenser) (n = 639/642)**				0.269	0.645	0.04	0.00-1.00
Strongly disagree	333 (52.1%)	98 (53.0%)	234 (51.9%)				
Disagree	116 (18.2%)	34 (18.4%)	81 (18.0%)				
Neutral	89 (13.9%)	18 (9.7%)	71 (15.7%)				
Agree	48 (7.5%)	17 (9.2%)	30 (6.7%)				
Strongly agree	53 (8.3%)	18 (9.7%)	35 (7.8%)				
**My veterinarian has informed me about possible side effects (n = 704/708)**				<0.001	<0.001	0.17	0.08-1.00
Strongly disagree	224 (31.8%)	53 (25.7%)	168 (34.0%)				
Disagree	110 (15.6%)	35 (17.0%)	75 (15.2%)				
Neutral	101 (14.3%)	18 (8.7%)	83 (16.8%)				
Agree	88 (12.5%)	23 (11.2%)	64 (13.0%)				
Strongly agree	181 (25.7%)	77 (37.4%)	104 (21.1%)				
**My vet told me exactly how to give the antibiotic (for example, how often, how many tablets/injections, how long) (n = 701/708)**				0.925	>0.999	0.00	0.00-1.00
Strongly disagree	10 (1.4%)	2 (1.0%)	8 (1.6%)				
Disagree	8 (1.1%)	2 (1.0%)	6 (1.2%)				
Neutral	34 (4.9%)	9 (4.4%)	25 (5.1%)				
Agree	88 (12.6%)	23 (11.2%)	64 (13.0%)				
Strongly agree	561 (80.0%)	169 (82.4%)	389 (79.1%)				
**I followed exactly the recommendations of my veterinarian for the antibiotic administration (n =699/708)**				0.601	0.901	0.00	0.00-1.00
Strongly disagree	5 (0.7%)	2 (1.0%)	3 (0.6%)				
Disagree	3 (0.4%)	2 (1.0%)	1 (0.2%)				
Neutral	7 (1.0%)	2 (1.0%)	5 (1.0%)				
Agree	37 (5.3%)	12 (5.9%)	25 (5.1%)				
Strongly agree	647 (92.6%)	187 (91.2%)	456 (93.1%)				
**I stuck to the exact number of tablets/capsules per antibiotic administration (n = 582/584)**				0.582	0.901	0.00	0.00-1.00
Strongly disagree	2 (0.3%)	1 (0.6%)	1 (0.2%)				
Disagree	1 (0.2%)	0 (0.0%)	1 (0.2%)				
Neutral	0 (0.0%)	0 (0.0%)	0 (0.0%)				
Agree	12 (2.1%)	2 (1.2%)	10 (2.5%)				
Strongly agree	567 (97.4%)	169 (98.3%)	395 (97.1%)				
**I stuck to the exact amount of solution/suspension/paste to be given per antibiotic administration (n = 38/38)**				>0.999	>0.999	0.00	0.00-1.00
Strongly disagree	0 (0.0%)	0 (0.0%)	0 (0.0%)				
Disagree	0 (0.0%)	0 (0.0%)	0 (0.0%)				
Neutral	1 (2.6%)	0 (0.0%)	1 (3.2%)				
Agree	0 (0.0%)	0 (0.0%)	0 (0.0%)				
Strongly agree	37 (97.4%)	7 (100.0%)	30 (96.8%)				
**I followed the exact number of antibiotic doses per day (n = 640/642)**				0.542	0.901	0.00	0.00-1.00

	Overall<br>n = 708	Medical training<br>n = 206	No medical training<br>n = 498	p-value[1]	q-value[2]	Cramer´s V[3]	95% CI[4]
Strongly disagree	2 (0.3%)	1 (0.5%)	1 (0.2%)				
Disagree	2 (0.3%)	0 (0.0%)	2 (0.4%)				
Neutral	2 (0.3%)	1 (0.5%)	1 (0.2%)				
Agree	16 (2.5%)	6 (3.2%)	10 (2.2%)				
Strongly agree	618 (96.6%)	179 (95.7%)	436 (96.9%)				
**I strictly followed the time intervals between antibiotic administrations (n = 636/642)**				0.067	0.267	0.00	0.00-1.00
Strongly disagree	1 (0.2%)	1 (0.5%)	0 (0.0%)				
Disagree	2 (0.3%)	0 (0.0%)	2 (0.4%)				
Neutral	35 (5.5%)	15 (8.0%)	20 (4.5%)				
Agree	186 (29.2%)	60 (32.1%)	124 (27.8%)				
Strongly agree	412 (64.8%)	111 (59.4%)	300 (67.3%)				
**I have suspended the administration of the antibiotic at least once because I was not able to administer it (n = 640/642)**				0.247	0.645	0.04	0.00-1.00
Strongly disagree	579 (90.5%)	164 (87.7%)	412 (91.6%)				
Disagree	19 (3.0%)	9 (4.8%)	10 (2.2%)				
Neutral	8 (1.3%)	4 (2.1%)	4 (0.9%)				
Agree	13 (2.0%)	3 (1.6%)	10 (2.2%)				
Strongly agree	21 (3.3%)	7 (3.7%)	14 (3.1%)				
**I always went to the vet for the injections on the scheduled appointment (n = 173/182)**				0.939	>0.999	0.00	0.00-1.00
Strongly disagree	14 (8.1%)	5 (10.6%)	9 (7.2%)				
Disagree	1 (0.6%)	0 (0.0%)	1 (0.8%)				
Neutral	4 (2.3%)	1 (2.1%)	3 (2.4%)				
Agree	4 (2.3%)	1 (2.1%)	3 (2.4%)				
Strongly agree	150 (86.7%)	40 (85.1%)	109 (87.2%)				
**I skipped an injection at least once and did not go to the vet (n = 177/182)**				>0.999	>0.999	0.00	0.00-1.00
Strongly disagree	173 (97.7%)	49 (100.0%)	123 (96.9%)				
Disagree	2 (1.1%)	0 (0.0%)	2 (1.6%)				
Neutral	1 (0.6%)	0 (0.0%)	1 (0.8%)				
Agree	0 (0.0%)	0 (0.0%)	0 (0.0%)				
Strongly agree	1 (0.6%)	0 (0.0%)	1 (0.8%)				

[1]Pearson's Chi-squared test; Fisher's exact test

[2]False discovery rate correction for multiple testing

[3]Interpretation: > 0.25 very strong, > 0.15 strong, > 0.10 moderate, > 0.05 weak, > 0 no or very weak association [27]

[4] CI = confidence interval of effect size

Table S9. Comparison between medical training and hand washing or wearing gloves.

	Overall n = 600	Medical training n = 178	No medical training n = 420	p-value[1]	q-value[2]	Cramer´s V[3]	95% CI[4]
**Did you wash your hands immediately after administering the antibiotic?**				0.014	0.014	0.11	0.00-1.00
Yes	336 (56.0%)	111 (62.4%)	224 (53.3%)				
No	247 (41.2%)	59 (33.1%)	187 (44.5%)				
No, but I wore gloves	4 (0.7%)	3 (1.7%)	1 (0.2%)				
Yes and I wore gloves	13 (2.2%)	5 (2.8%)	8 (1.9%)				

[1]Fisher's exact test

[2]False discovery rate correction for multiple testing

[3]Interpretation: > 0.25 very strong, > 0.15 strong, > 0.10 moderate, > 0.05 weak, > 0 no or very weak association [27]

[4] CI = confidence interval of effect size

Table S10: Adverse reactions and premature discontinuation of administration

**Did you stop antibiotic administration before the duration of use indicated by your veterinarian was completed? (n = 706/708)**	Yes	29/706 (4.1%)
	No	677/706 (95.9%)
**What was the reason for stopping the administration early? (Multiple answers possible)**	Difficulties with ingestion/application	7/29 (24.1%)
	Lack of time	0
	Side effects have occurred	9/29 (31.0%)
	Fear of potential side effects	2/29 (6.9%)
	My dog/cat had already recovered	2/29 (6.9%)
	Consultation with my veterinarian	8/29 (27.6%)
	Other reason	5/29 (17.2%)
**Did your animal experience any side effects that may have occurred as a result of the administration of the antibiotic? (n = 622/708)**	Yes	145/622 (21.9%)
	No	517/622 (78.1%)
**What side effects have occurred? (Multiple answers possible)**	Diarrhea	87/145 (60.0%)
	Vomiting	25/145 (17.2%)
	Allergic reaction	25/145 (17.2%)
	Other side effect	51/145 (35.2%)

Table S11: Culture and sensitivity testing before antibiotic administration

**Did your veterinarian perform any tests prior to administering the antibiotic, based on the results of which the antibiotic was prescribed/administered? (Multiple answers possible)**	No	364/708 (51.4%)
	Yes, there were changes in the blood count	97/708 (13.7%)
	Yes, there were changes in the X-ray images	26/708 (3.7%)
	Yes, changes were present in the urine analysis	54/708 (7.6%)
	Yes, changes were present in the ultrasound examination	41/708 (5.8%)
	Yes, changes were present in a microscopic examination	71/708 (10.0%)
	Yes, changes were present in other examinations	124/708 (17.5%)
	Yes, but i do not know what test or changes exactly	17/708 (2.4%)
	I do not know	31/708 (4.4%)
**Was a culture with resistance test or other tests for pathogen detection performed prior to antibiotic administration/prescription? (n = 648/708)**	Yes	125/648 (19.3%)
	No	523/648 (80.7%)

Table S12: Comparison between antibiotic and C&S.

	**Overall n = 472**	**C&S n = 106**	**No C&S n = 366**	**p-value[1]**	**q-value[2]**	**Cramer´s V[3]**	**95% CI[4]**
				< 0.001	< 0.001	0.20	0.12-1.00
Kein Reserveantibiotikum (Other antibiotics)	430 (100.0%)	85 (19.8%)	345 (80.2%)				
Reserveantibiotikum (Fluorochinolones and 3rd generation cephalosporins)	42 (100.0%)	21 (50.0%)	21 (50.0%)				

[1]Pearson's Chi-squared test

[2]False discovery rate correction for multiple testing

[3]Interpretation: > 0.25 very strong, > 0.15 strong, > 0.10 moderate, > 0.05 weak, > 0 no or very weak association [27]

[4] CI = confidence interval of effect size

Table S13: Comparison between reason and C&S.

	Overall n= 648	C&S n = 125	No C&S n = 523	p-value[1]	q-value[2]	Cramer`s V[3]	95% CI[4]
Diarrhea	100 (15.4%)	20 (16.0%)	80 (15.3%)	0.890	> 0.999	0.00	0.00-1.00
Vomiting	55 (8.5%)	6 (4.8%)	49 (9.4%)	0.110	0.439	0.05	0.00-1.00
Infection of the upper respiratory tract	59 (9.1%)	12 (9.6%)	47 (9.0%)	0.863	> 0.999	0.00	0.00-1.00
Skin problems	47 (7.3%)	10 (8.0%)	37 (7.1%)	0.703	> 0.999	0.00	0.00-1.00
Ear infection	46 (7.1%)	17 (13.6%)	29 (5.5%)	0.003	0.019	0.12	0.04-1.00
Inffection of the lower respiratory tract	53 (8.2%)	14 (11.2%)	39 (7.5%)	0.202	0.538	0.04	0.00-1.00
Fever	43 (6.6%)	7 (5.6%)	36 (6.9%)	0.693	> 0.999	0.00	0.00-1.00
Wound infection/abscess/bite injury	127 (19.6%)	21 (16.8%)	106 (20.3%)	0.452	> 0.999	0.00	0.00-1.00
Castration/neutering	38 (5.9%)	6 (4.8%)	32 (6.1%)	0.676	> 0.999	0.00	0.00-1.00
Surgery of the bones	26 (4.0%)	2 (1.6%)	24 (4.6%)	0.201	0.538	0.05	0.00-1.00
Surgery on soft tissue	58 (9.0%)	11 (8.8%)	47 (9.0%)	> 0.999	> 0.999	0.00	0.00-1.00
Dental treatment	79 (12.2%)	6 (4.8%)	73 (14.0%)	0.004	0.019	0.10	0.02-1.00
Other surgery	10 (1.5%)	2 (1.6%)	8 (1.5%)	> 0.999	> 0.999	0.00	0.00-1.00
Urinary tract infection	62 (9.6%)	27 (21.6%)	35 (6.7%)	< 0.001	< 0.001	0.20	0.13-1.00
Eye problem	32 (4.9%)	6 (4.8%)	26 (5.0%)	> 0.999	> 0.999	0.00	0.00-1.00
Other problem	88 (13.6%)	15 (12.0%)	73 (14.0%)	0.663	> 0.999	0.00	0.00-1.00

[1]Fisher's exact test

[2]False discovery rate correction for multiple testing
[3]Interpretation: > 0.25 very strong, > 0.15 strong, > 0.10 moderate, > 0.05 weak, > 0 no or very weak association [27]
[4]CI = confidence interval of effect size

Table S14: 5-point Likert scale regarding preferred route for systemic antibiotic administration comparing dogs and cats.

	Overall n = 708	Dog n = 463	Cat n = 245	p-value[1]	q-value[2]	Cramer´s V[3]	95% CI[4]
**Tablets**				< 0.001	< 0.001	0.28	0.21-1.00
Not at all	51 (7.2%)	21 (4.5%)	30 (12.2%)				
Little	39 (5.5%)	11 (2.4%)	28 (11.4%)				
Neutral	72 (10.2%)	35 (7.6%)	37 (15.1%)				
Strong	175 (24.7%)	119 (25.7%)	56 (22.9%)				
Very strong	371 (52.4%)	277 (59.8%)	94 (38.4%)				
**Capsules**				< 0.001	< 0.001	0.26	0.18-1.00
Not at all	130 (18.4%)	62 (13.4%)	68 (27.8%)				
Little	97 (13.7%)	49 (10.6%)	48 (19.6%)				
Neutral	174 (24.6%)	112 (24.2%)	62 (25.3%)				
Strong	132 (18.6%)	99 (21.4%)	33 (13.5%)				
Very strong	175 (24.7%)	141 (30.5%)	34 (13.9%)				
**Solution/suspension/paste**				0.010	0.014	0.11	0.00-1.00
Not at all	172 (24.3%)	123 (26.6%)	49 (20.0%)				
Little	112 (15.8%)	77 (16.6%)	35 (14.3%)				
Neutral	140 (19.8%)	97 (21.0%)	43 (17.6%)				
Strong	126 (17.8%)	67 (14.5%)	59 (24.1%)				
Very strong	158 (22.3%)	99 (21.4%)	59 (24.1%)				

	Overall n = 708	Dog n = 463	Cat n = 245	p-value[1]	q-value[2]	Cramer´s V[3]	95% CI[4]
**Short-acting injection**				0.011	0.014	0.11	0.00-1.00
Not at all	200 (28.2%)	148 (32.0%)	52 (21.2%)				
Little	147 (20.8%)	89 (19.2%)	58 (23.7%)				
Neutral	141 (19.9%)	94 (20.3%)	47 (19.2%)				
Strong	105 (14.8%)	58 (12.5%)	47 (19.2%)				
Very strong	115 (16.2%)	74 (16.0%)	41 (16.7%)				
**Long-acting injection**				0.019	0.019	0.10	0.00-1.00
Not at all	142 (20.1%)	100 (21.6%)	42 (17.1%)				
Little	75 (10.6%)	50 (10.8%)	25 (10.2%)				
Neutral	122 (17.2%)	86 (18.6%)	36 (14.7%)				
Strong	154 (21.8%)	106 (22.9%)	48 (19.6%)				
Very strong	215 (30.4%)	121 (26.1%)	94 (38.4%)				

[1]Pearson's Chi-squared test
[2]False discovery rate correction for multiple testing
[3]Interpretation: > 0.25 very strong, > 0.15 strong, > 0.10 moderate, > 0.05 weak, > 0 no or very weak association [27]
[4] CI = confidence interval of effect size

Table S15: Tablet administration

	Overall n = 706	Dog n = 462	Cat n = 244	p-value[1]	q-value[2]	Cramer´s V[3]	95% IC
**How well does your dog/cat take tablets?**				< 0.001	< 0.001	0.32	0.25-1.00
Not at all	29 (4.1%)	8 (1.7%)	21 (8.6%)				
Rather less good	67 (9.5%)	24 (5.2%)	43 (17.6%)				
Neutral	75 (10.6%)	38 (8.2%)	37 (15.2%)				
Rather good	141 (20.0%)	88 (19.0%)	53 (21.7%)				
Very good	394 (55.8%)	304 (65.8%)	90 (36.9%)				
	**Overall n = 576**	**Dog n = 360**	**Cat n = 216**	**p-value[1]**	**q-value[2]**	**Cramer´s V[3]**	**95% CI[4]**
**Does your dog/cat tolerate liquid/paste medication (solution/suspension/paste) better than tablets/capsules?**				< 0.001	< 0.001	0.18	0.11-1.00
Yes	154 (26.7%)	73 (20.3%)	81 (37.5%)				
No	422 (73.3%)	287 (79.7%)	135 (62.5%)				
	**Overall n = 567**	**Dog n = 390**	**Cat n = 177**	**p-value[1]**	**q-value[2]**	**Cramer´s V[3]**	**95% CI[4]**
**Is your pet`s ability to take tablets related to the taste of the medication?**				0.003	0.003	0.12	0.04-1.00
Yes	289 (51.0%)	182 (46.7%)	107 (60.5%)				
No	278 (49.0%)	208 (53.3%)	70 (39.5%)				

[1]Pearson's Chi-squared test
[2]False discovery rate correction for multiple testing
[3]Interpretation: > 0.25 very strong, > 0.15 strong, > 0.10 moderate, > 0.05 weak, > 0 no or very weak association [27]
[4]CI = confidence interval of effect size

Table S16: 5-point Likert scale regarding influencing factors on antibiotic administration comparing dogs and cats.

	Overall n = 708	Dog n = 463	Cat n = 245	p-value[1]	q-value[2]	Cramer´s V[3]	95% CI[4]
**Easy form of administration**				0.001	0.004	0.14	0.03-1.00
Not at all important	54 (7.6%)	40 (8.6%)	14 (5.7%)				
Not very important	45 (6.4%)	35 (7.6%)	10 (4.1%)				
Somewhat important	111 (15.7%)	85 (18.4%)	26 (10.6%)				
Very important	175 (24.7%)	115 (24.8%)	60 (24.5%)				
Extremely important	323 (45.6%)	188 (40.6%)	135 (55.1%)				
**Costs**				0.004	0.007	0.13	0.00-1.00
Not at all important	200 (28.2%)	116 (25.1%)	84 (34.3%)				
Not very important	165 (23.3%)	100 (21.6%)	65 (26.5%)				
Somewhat important	216 (30.5%)	152 (32.8%)	64 (26.1%)				
Very important	86 (12.1%)	61 (13.2%)	25 (10.2%)				
Extremely important	41 (5.8%)	34 (7.3%)	7 (2.9%)				
**Tolerability (potential side effects)**				0.002	0.004	0.13	0.00-1.00
Not at all important	1 (0.1%)	1 (0.2%)	0 (0.0%)				
Not very important	2 (0.3%)	2 (0.4%)	0 (0.0%)				
Somewhat important	34 (4.8%)	13 (2.8%)	21 (8.6%)				
Very important	159 (22.5%)	97 (21.0%)	62 (25.3%)				
Extremely important	512 (72.3%)	350 (75.6%)	162 (66.1%)				
**Avoiding the development of antibiotic resistance**				0.105	0.168	0.07	0.00-1.00
Not at all important	4 (0.6%)	3 (0.6%)	1 (0.4%)				
Not very important	9 (1.3%)	8 (1.7%)	1 (0.4%)				
Somewhat important	56 (7.9%)	30 (6.5%)	26 (10.6%)				
Very important	157 (22.2%)	97 (21.0%)	60 (24.5%)				
Extremely important	482 (68.1%)	325 (70.2%)	157 (64.1%)				
**Efficacy**				0.684	0.684	0.00	0.00-1.00
Not at all important	0 (0.0%)	0 (0.0%)	0 (0.0%)				
Not very important	0 (0.0%)	0 (0.0%)	0 (0.0%)				
Somewhat important	1 (0.1%)	1 (0.2%)	0 (0.0%)				
Very important	44 (6.2%)	31 (6.7%)	13 (5.3%)				
Extremely important	663 (93.6%)	431 (93.1%)	232 (94.7%)				
**Recommendation of my veterinarian**				0.495	0.565	0.00	0.00-1.00
Not at all important	10 (1.4%)	7 (1.5%)	3 (1.2%)				
Not very important	22 (3.1%)	12 (2.6%)	10 (4.1%)				
Somewhat important	110 (15.5%)	76 (16.4%)	34 (13.9%)				
Very important	275 (38.8%)	172 (37.1%)	103 (42.0%)				
Extremely important	291 (41.1%)	196 (42.3%)	95 (38.8%)				
**Tastefulness of the antibiotic**				<0.001	<0.001	0.25	0.18-1.00
Not at all important	156 (22.0%)	129 (27.9%)	27 (11.0%)				
Not very important	126 (17.8%)	98 (21.2%)	28 (11.4%)				
Somewhat important	223 (31.5%)	129 (27.9%)	94 (38.4%)				
Very important	122 (17.2%)	63 (13.6%)	59 (24.1%)				
Extremely important	81 (11.4%)	44 (9.5%)	37 (15.1%)				
**Avoidance of „reserve antibiotics“**				0.307	0.409	0.03	0.00-1.00
Not at all important	23 (3.2%)	15 (3.2%)	8 (3.3%)				
Not very important	32 (4.5%)	18 (3.9%)	14 (5.7%)				
Somewhat important	145 (20.5%)	98 (21.2%)	47 (19.2%)				

	**Overall n = 708**	**Dog n = 463**	**Cat n = 245**	**p-value[1]**	**q-value[2]**	**Cramer´s V[3]**	**95% CI[4]**
Very important	192 (27.1%)	116 (25.1%)	76 (31.0%)				
Extremely important	316 (44.6%)	216 (46.7%)	100 (40.8%)				

[1]Pearson's Chi-squared test; Fisher's exact test

[2]False discovery rate correction for multiple testing

[3]Interpretation: > 0.25 very strong, > 0.15 strong, > 0.10 moderate, > 0.05 weak, > 0 no or very weak association [27]

[4] CI = confidence interval of effect size
